# Artificial intelligence in the radiological diagnosis of cancer

**DOI:** 10.6026/9732063002001512

**Published:** 2024-09-30

**Authors:** Bassam Alkhalifah

**Affiliations:** 1 Department of Radiology and Medical Imaging, College of Medicine, Qassim University, Buraydah, Saudi Arabia

**Keywords:** Artificial intelligence, cancer, detection, deep learning, prediction, sensitivity

## Abstract

Artificial intelligence (AI) is being used to diagnose deadly diseases such as cancer. The possible decrease in human error, fast diagnosis, and consistency of judgment are the key incentives for implementing these technologies. Therefore, it is of interest to assess the use of artificial intelligence in cancer diagnosis. Total 200 cancer cases were included with 100 cases each of Breast and lung cancer to evaluate with AI and conventional method by the radiologist. The cancer cases were identified with the application of AI-based machine learning techniques. The sensitivity and specificity check-up was used to assess the effectiveness of both approaches. The obtained data was statistically evaluated. AI has shown higher accuracy, sensitivity and specificity in cancer diagnosis compared to manual method of diagnosis by radiologist.

## Background:

Since cancer is the major cause of death worldwide, accurate cancer diagnosis is essential [1]. The use of imaging technologies to detect cancer early helps people save money and lives by reducing the need for treatment. A vital component of cancer care is radiology. Imaging, a non-invasive technique, may evaluate all of the methods used to identify cancer early on and determine its prognosis, including computed tomography (CT), magnetic resonance imaging (MRI), ultrasound, and X-rays. The F-fluorodeoxyglucose is used in the positron emission tomography technique [1, 2]. In order to investigate the interior structure of the body, sound waves are delivered inside it using the ultrasound technique. This method makes use of a transducer. Tissues are viewed as barriers by them, and they respond by echoing back and it records these echoes. For digital analysis, the echo values are subsequently converted to greyscale [1]. For an informed diagnosis, accurate identification requires the mining of quantitative data. Conventional picture interpretation techniques can be laborious, prone to human mistake, and worsened by complex errors [1]. Radiologists employ images of cancer in various stages of reading, analysing, and diagnosing the disease. But their heavy job and long working hours can result in poor judgement, incorrect diagnosis, or missing diagnoses. Potential human errors can be reduced by using AI, or in this case, computer-aided diagnosis (CAD) [3]. AI is one of the fields of computers and informatics that is expanding the fastest and it has a lot to do with radiology and healthcare. When it comes to creating and evaluating AI applications for medical imaging, radiologists need to take the lead [4]. While machine learning (ML) is a subset of artificial intelligence (AI), machine learning (ML) is the process of using data to create predictions or classifications, either with or without human supervision [5]. AI is defined as the construction of robots or systems that can replicate human thought and conduct. The growing prevalence of artificial intelligence (AI) methods has opened up significant opportunities for their use in a number of medical domains, including prognosis, intelligent rehabilitation, in vitro diagnosis and medical imaging [3]. The amount of time it takes to diagnose a patient can be greatly decreased by using AI, which can analyse and interpret images much more quickly. Artificial intelligence (AI) algorithms are able to spot patterns and abnormalities that the human eye might miss by analysing large databases of medical images [6].

The field of diagnostic imaging in healthcare is undergoing a major transformation because of artificial intelligence (AI). This technique is a significant development in the interpretation and use of medical images, such as X-rays, MRIs, and CT scans, as it combines complex algorithms and machine learning [7]. Artificial Intelligence (AI) is the ability of a computer system to precisely comprehend and learn from outside inputs and to modify knowledge to perform certain jobs in a flexible manner [3]. AI enables early disease detection by analysing historical data to find trends or risk factors. AI can offer customised insights, resulting in more individualised and successful treatment regimens [7]. Numerous scholars have carried out investigations and devised techniques to evaluate the efficacy of a specific cancer detection device. AI has created improvements in several medical sectors, including diagnosis, therapy, medication development, patient care, *etc.* Numerous research projects have been started on AI's use in cancer diagnosis. AI-based technologies assist radiotherapists to become more skilled investigators and aid in the early diagnosis of various tumours [1].

## AI and ML techniques in cancer imaging:

In the field of cancer imaging, patient images are obtained, pre-processed, and transformed as inputs for machine learning algorithms and models (to guarantee data conformance or consistency). Whether they are related to features defined by radiologists or features generated statistically from radiomics, such pre-processing processes are employed. This entails making certain that the photos have comparable pixel sizes and image section thicknesses. In summary, a machine learning model or algorithm maps the input imaging data and learns a mathematical function, either basic or complex, that is associated with the target or output, which could be anything from a scientific or clinical observation. Any machine learning algorithm's likelihood of success depends on the availability of data, the processing capacity of the machine, and further algorithm improvements. With larger datasets, more complex ML models, such as convolutional neural networks (CNN) that is particularly efficient in learning directly from images [5].

When it comes to clinical decision-making, artificial intelligence can improve things by measuring information from imagery that is not visible to humans. AI can also make it possible to combine many data sources to create strong integrated diagnostic systems [8]. AI assists in cancer detection, staging, and monitoring by utilising prior data sets. Reconstruction techniques based on DLR have reduced radiation exposure and/or enhanced image quality while providing a respectably quick recovery time. Machine learning (ML) is useful to "precision cancer treatment" for treating cancer patients. "Precision cancer treatment" aims to accurately predict the right medication treatments for a particular patient based on the distinct genetic profiles of their tumours [9]. To mimic human intelligence, deep learning (DL), a subfield of machine learning, is used in drug development, identification and diagnostics. It uses an Artificial Neural Network (ANN) to simulate how input is processed by artificial neurons [10].

A unique kind of machine learning architecture as convolutional neural networks features distinct layers arranged within a kernel. In contrast to traditional computer-aided diagnosis (CAD) in convolutional neural networks, the algorithm itself determines during training which elements of the image are suggestive of the presence of a lesion. In contrast, the programmer's input is used in CAD [11].

One prevalent illness that has a serious impact on women's physical and emotional well-being is breast cancer. Patients with breast cancer can significantly improve their prognosis with early breast cancer screening using mammography, ultrasound, or magnetic resonance imaging (MRI). Currently the greatest cause of death for women worldwide, breast cancer is one of the most prevalent cancers found in women [3]. It has been demonstrated that mammography is one of the most effective breast cancer screening methods. AI-based methods for detecting breast cancer make use of machine learning and artificial intelligence capabilities. These algorithms identify the irregularities and patterns in the pictures. AI-based technologies assist in the early detection of breast cancer and enable radiotherapists become more proficient investigators.

Due to the disease's late stage at diagnosis, the majority of individuals with lung cancer will pass away from it. Computed tomography (CT) scans and lung radiography are the most widely used methods for identifying lung cancer [1, 12]. AI and medical imaging are anticipated to be key factors in enhancing lung cancer early identification and characterisation [8]. AI-based methods for the early identification and diagnosis of lung cancer analyse medical data, such as CT scans, chest X-rays and PET scans, using sophisticated machine learning algorithms. With an accuracy of 99.51%, the best result is obtained when deep features are used with KNN and MRMR [1]. There are very limited studies on comparison of AI with radiologist in cancer detection. Therefore, it is of interest to compare the accuracy of AI technology over radiologist in cancer detection.

## Materials and Method:

This *in vivo* comparative study was done in the department of Radiology after obtaining the approval from concern authority and consent from all the participants. Total 200 cancer cases were included with 100 cases each of Breast and lung cancer to evaluate with AI and only conventional method (by radiologist). The cancer cases were identified with the application of AI-based machine learning techniques. The sensitivity and specificity check-up was used to assess the effectiveness of both approaches. An analysis by Singh *et al.* was conducted using an AI method for cancer diagnosis [1]. Architecture for a deep neural network was created. For fine-grained classification, it united the benefits of pre-trained features with a multi-resolution picture analysis using a feature pyramid network. The first feature extractors were specifically VGGNet (16 layers) and ResNet (50 layers), which were trained on millions of photos from Image Net. Convergence was enhanced by dilated blocks made of multiscale features and skip connections, while overfitting was decreased by spatial dropout [13]. The LOM-based area under the receiver operating characteristic curve (AUROC) and recall-based specificity and sensitivity were used to assess the performance of AI and radiologists. Deep learning is a popular type of artificial intelligence that is used in the recently developed AI-CAD for mammography called cmAssist. The cmAssist approach achieves high sensitivity without sacrificing specificity by combining many distinct deep learning-based networks. An array of connected neurones arranged into three layers-input, hidden and output-makes up an ANN-based architecture [1]. The obtained data was statistically evaluated using SPSS software version 23.0 IBM USA with P<0.05.

## Result:

Table 1 indicates the comparative evaluation of cancer detection using AI technology and by radiologist for breast and lung cancer. The result was statistically highly significant indicating that AI technique has more sensitivity and specificity in detection. Table 2 indicates the breast cancer detection using conventional method over AI method and it indicates that, AI is more accurate than convention technique.

## Discussion:

AI is being used for cancer detection more and more [1]. AI helps physicians treat patients with greater precision and efficacy [14]. There is a lot of interest in applying artificial intelligence (AI) technology to image recognition-based cancer screening and detection since early diagnosis is linked to improved treatment outcomes for the patient. It's common to hear the phrases computer-aided diagnosis (CADx) and computer-aided detection (CADe) [15]. It has been demonstrated that machine learning (ML), a subfield of artificial intelligence (AI), reduces the likelihood of dysplasia and cancer classification errors, ensuring consistency and validity and impacting treatment choices [15]. Form the present study we found that AI has higher specificity and accuracy compared to manual method in detection of breast and lung cancer. The difference was highly significant. A systematic review by Khalifa *et al.* assessed how artificial intelligence (AI) is changing diagnostic imaging in the medical field. They came to the conclusion that, by enhancing accuracy and efficiency, AI is revolutionising diagnostic imaging [7]. The effectiveness of artificial intelligence (AI) algorithms in practical radiology processes was evaluated by Wu *et al.* They comprised 72 conclusions compiled by medical specialists to provide a comprehensive preliminary reading of AP frontal chest radiographs. According to their suggestion, it is feasible to create AI systems for full-fledged preliminary readings of AP frontal chest radiographs that both match and surpass the mean performance level of third-year radiology residents [13]. When it comes to identifying breast cancer in mammograms, an AI system built on deep learning algorithms performs comparably to an average radiologist [16]. Batool *et al.* using artificial intelligence based classifiers and optimized feature reduction technique assessed for breast cancer diagnosis. They came to the conclusion that Relief had proven to be incredibly effective, with a maximum accuracy of 98.2% [17]. The Lung Images Dataset Consortium and Image Dataset Resource Initiative (LIDC-IDRI) datasets were utilised by Sasikala *et al.* to diagnose lung cancer. 96% accuracy was attained [18]. Sharif *et al.* discovered that AI could diagnose breast cancer with 93.5% accuracy [19]. Based on deep fusion learning, Yu *et al.* discovered 87% accuracy in breast cancer diagnosis [20]. These outcomes support our conclusions regarding accuracy.

The accuracy with which Artificial Intelligence (AI) approaches are applied in the identification and diagnosis of malignant tumours in adult patients was examined by da Silva *et al.* They concluded that, the detection and diagnosis of malignant tumours with the help of AI seems to be feasible and accurate with the use of different technologies [15]. Based on a thorough evaluation, Derevianko *et al.* came to the conclusion that AI can assist physicians in diagnosing patients [4]. Dong Z *et al.* evaluated a real-time artificial intelligence (AI) system that distinguished between leiomyoma's and GISTs based on endoscopic ultrasound (EUS) pictures. They came to the conclusion that, in clinical practice, endoscopists can be helped to quickly and reliably differentiate between different types of SELs by including a real-time AI system during EUS tests [21]. In a study by Nindrea *et al.* K-Nearest Neighbour (KNN), artificial neural network (ANN), decision tree (DT), and Naïve Bayes (NB) were the four additional classification techniques that were compared to support vector machine (SVM). In the breast cancer risk calculation, SVM was demonstrated to yield the best area under the curve (AUC), with AUC > 90%. The accuracy rate of the SVM is 97.13% [22]. The X2GAI model by Oztekin *et al.* was compared based on the biopsy's diagnosis. When seasoned radiologists reported false positive results, it was noted that the new model performed well [23].

## Limitation of AI:

The application of AI in diagnostic imaging presents a variety of challenging and ethical issues that should be carefully explored, despite its apparent promise. Further research is needed on larger sample size with geographical assessment.

## Conclusion:

AI is a fast and effective method for diagnosis.AI has shown higher accuracy, sensitivity and specificity in cancer diagnosis. Radiologist can utilise the AI in detection of cancer for better interpretation and result.

## Figures and Tables

**Table 1:** Comparative assessment of cancer detection accuracy using AI

**Detection of Cancer types**		**Accuracy in detection (Mean ±SD)**		p
		**Group I-AI performance**	**Group II- radiologist observation**	
Brest cancer	sensitivity	92%	73%	0.001
	T 1 tumour detection	91%	69%	
	specificity	92%	74%	
Lung cancer	sensitivity	93%	71%	0.001
	Specificity	90%	67%	

**Table 2:** Breast cancer detection with different techniques

**Detection method**	**Accuracy in percentage**	p
Conventional Auto Encoder	92%	0.05
Artificial intelligence (ANN)	97%	
ANN with extreme learning	95%	
